# Supplementary material for: Telomere-to-mitochondria signalling by ZBP1 mediates replicative crisis
Source: Nature. 2023 Feb 8;614(7949):767–73. doi: 10.1038/s41586-023-05710-8 (PMC9946831; doi:10.1038/s41586-023-05710-8)
Supplement: Supplementary file 1 — Supplementary Figs. 1–6. Supplementary Figs. 1–5 contain the raw, uncropped images of the western blots, southern blots of terminal restriction fragments and RNA dot-blots. The molecular mass markers shown throughout this document are in kDa. Supplementary Fig. 6 shows a list of enriched gRNAs (log2 fold change > 2) of the CRISPR–Cas9 screen. Data represent the log2-transformed fold change of read counts before (day 0) and after enrichment (day 15). [file 41586_2023_5710_MOESM1_ESM.pdf]

nature portfolio

https://doi.org/10.1038/s41586-023-05710-8

Supplementary information

Telomere-to-mitochondria signalling by ZBP1 mediates replicative crisis

In the format provided by the authors and unedited

SI Figure 1

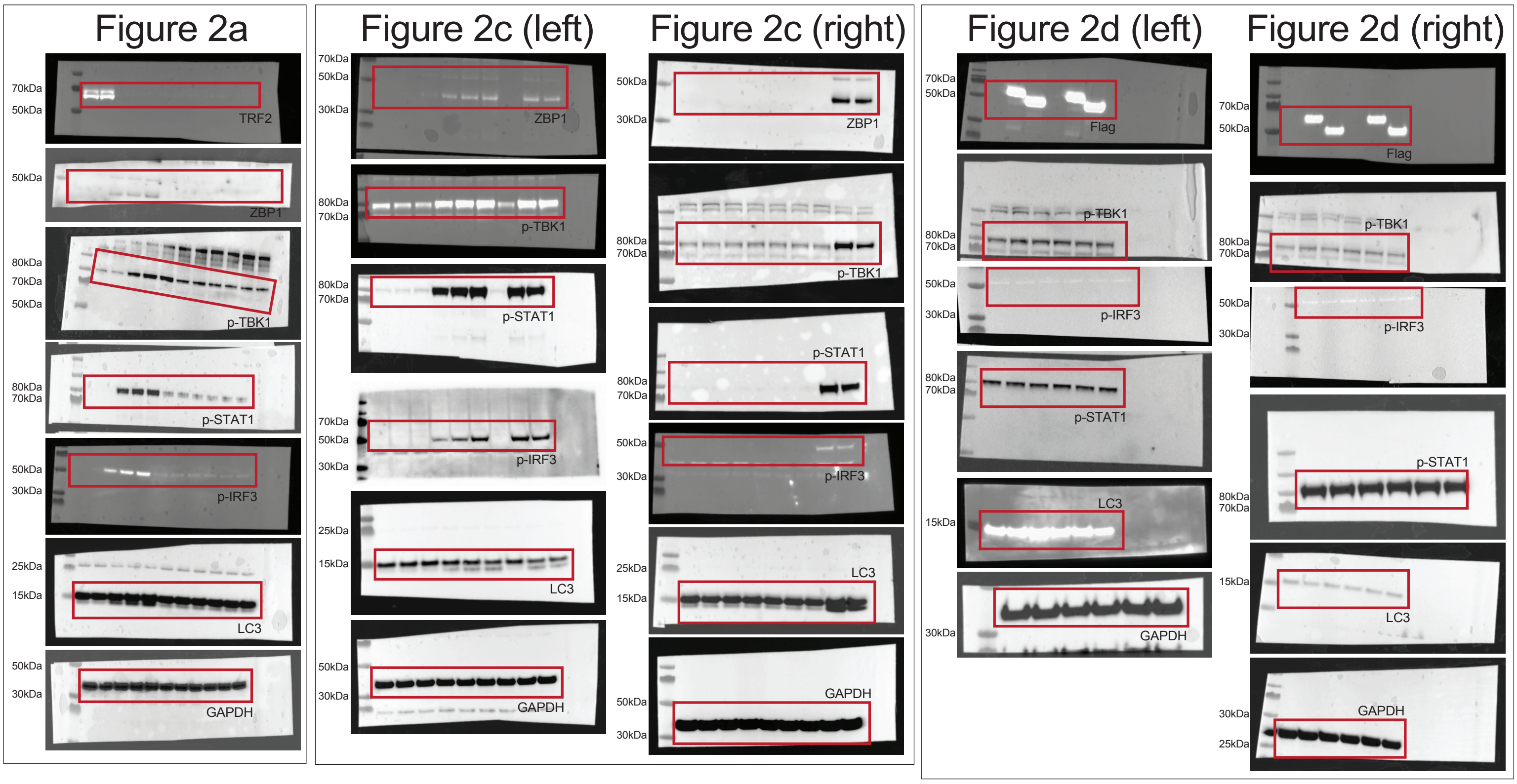
Figure 2a
70kDa
50kDa
TRF2
50kDa
ZBP1
80kDa
70kDa
50kDa
p-TBK1
80kDa
70kDa
p-STAT1
50kDa
30kDa
p-IRF3
25kDa
15kDa
LC3
50kDa
30kDa
GAPDH
Figure 2c (left)
70kDa
50kDa
30kDa
ZBP1
80kDa
70kDa
p-TBK1
80kDa
70kDa
p-STAT1
70kDa
50kDa
30kDa
p-IRF3
25kDa
15kDa
LC3
50kDa
30kDa
GAPDH
Figure 2c (right)
50kDa
30kDa
ZBP1
80kDa
70kDa
p-TBK1
p-STAT1
80kDa
70kDa
50kDa
30kDa
p-IRF3
25kDa
15kDa
LC3
GAPDH
50kDa
30kDa
Figure 2d (left)
70kDa
50kDa
Flag
p-TBK1
80kDa
70kDa
50kDa
30kDa
p-IRF3
80kDa
70kDa
p-STAT1
15kDa
LC3
30kDa
GAPDH
Figure 2d (right)
70kDa
50kDa
Flag
p-TBK1
80kDa
70kDa
50kDa
30kDa
p-IRF3
p-STAT1
80kDa
70kDa
15kDa
LC3
30kDa
25kDa
GAPDH

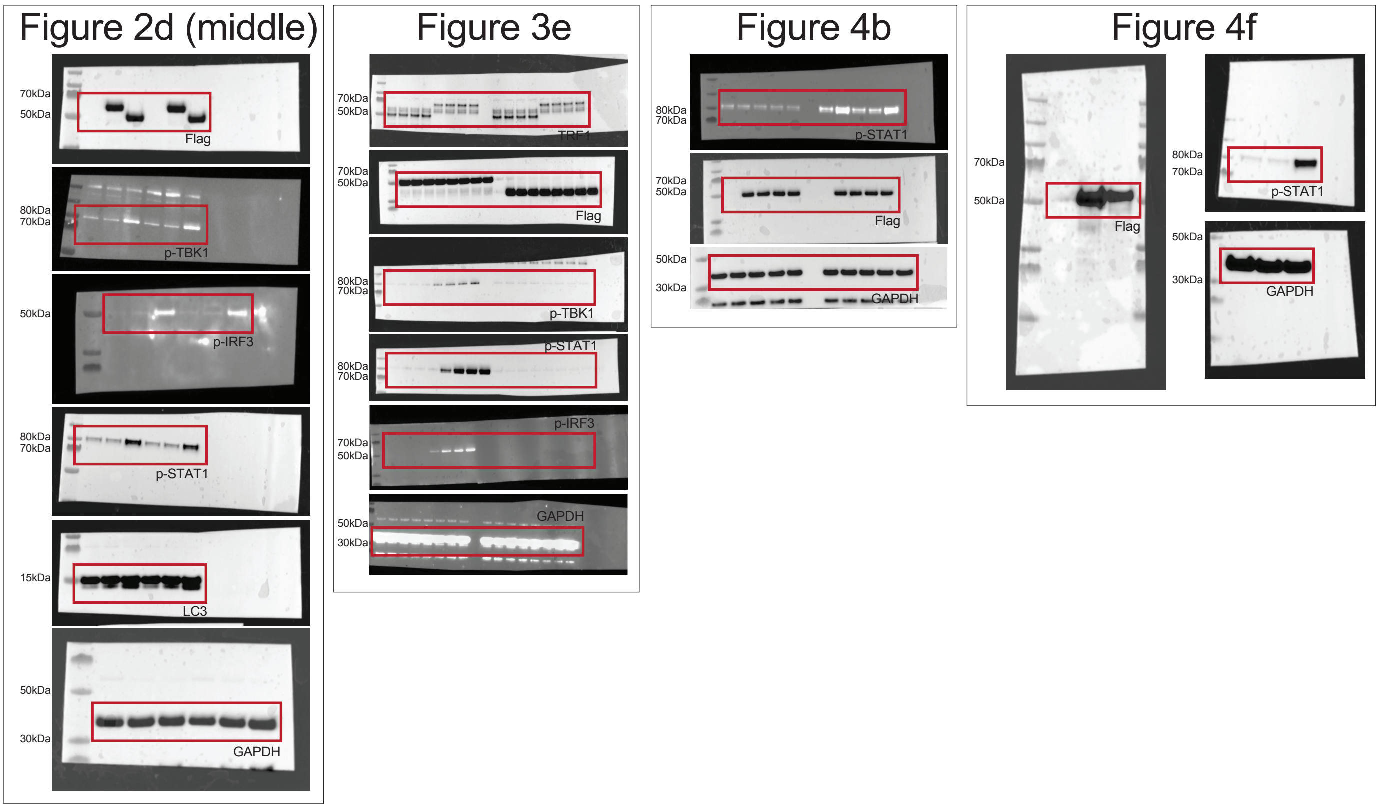
Figure 2d (middle)
70kDa
50kDa
Flag
80kDa
70kDa
p-TBK1
50kDa
p-IRF3
80kDa
70kDa
p-STAT1
15kDa
LC3
50kDa
30kDa
GAPDH
Figure 3e
70kDa
50kDa
TRF1
70kDa
50kDa
Flag
80kDa
70kDa
p-TBK1
p-STAT1
80kDa
70kDa
p-IRF3
70kDa
50kDa
GAPDH
50kDa
30kDa
Figure 4b
80kDa
70kDa
p-STAT1
70kDa
50kDa
Flag
50kDa
30kDa
GAPDH
Figure 4f
70kDa
50kDa
Flag
80kDa
70kDa
p-STAT1
50kDa
30kDa
GAPDH

SI Figure 2

ED Figure 3c

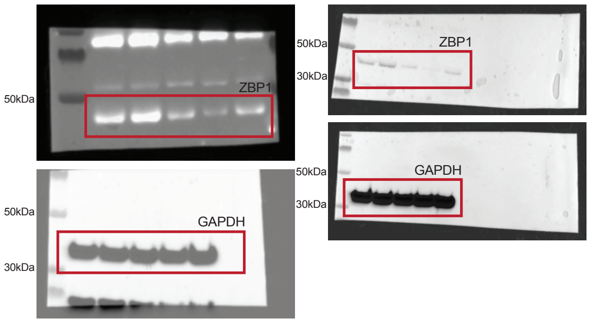

ED Figure 4c (left)

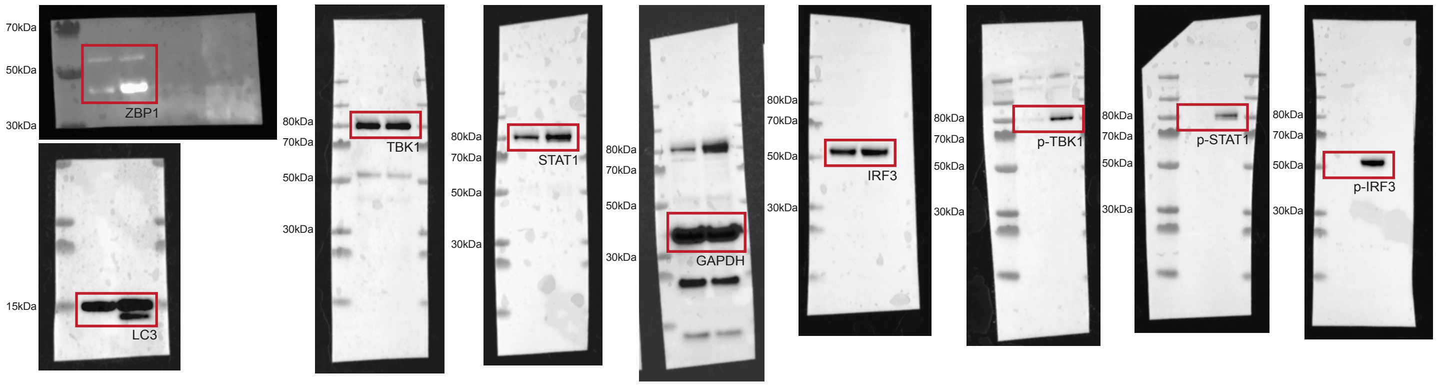

ED Figure 4c (right)

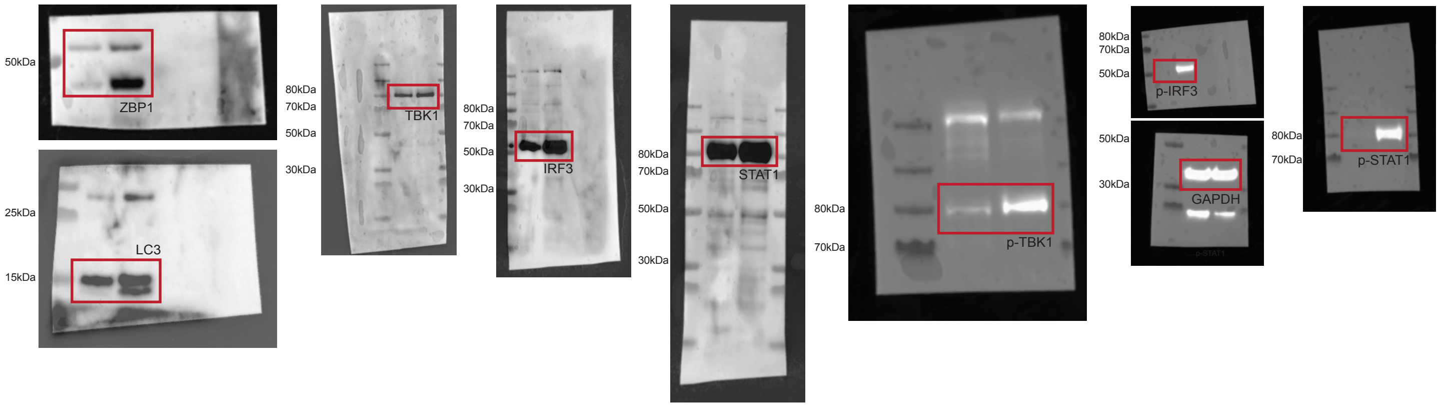

ED Figure 4d

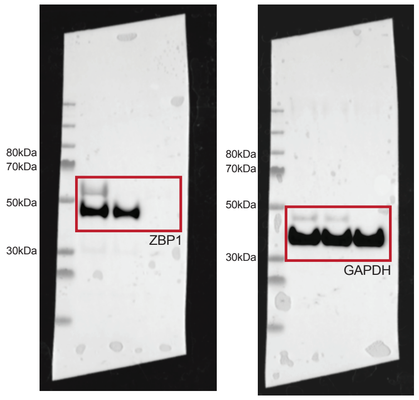

SI Figure 3

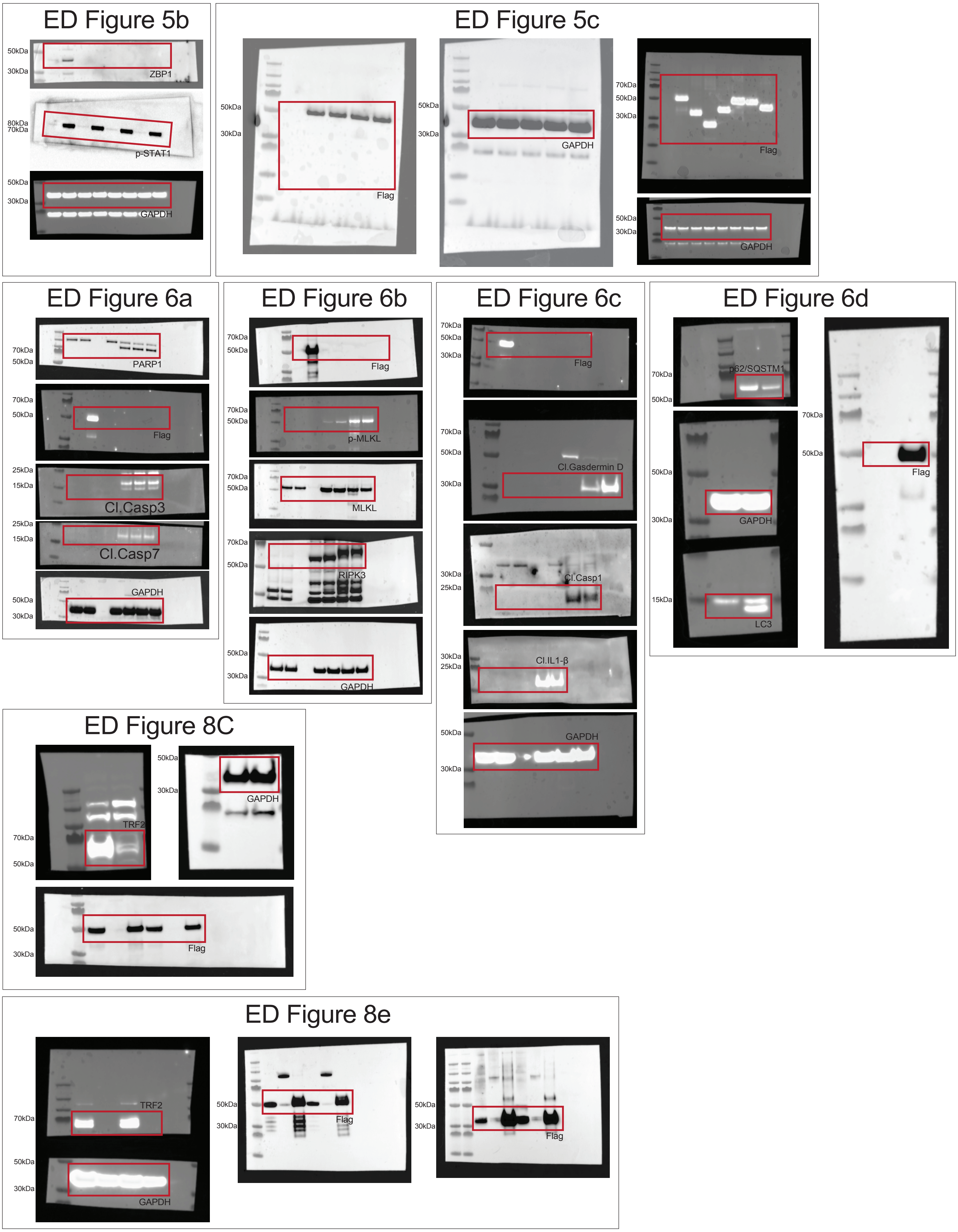
ED Figure 5b
50kDa
30kDa
ZBP1
80kDa
70kDa
p-STAT1
50kDa
30kDa
GAPDH
ED Figure 5c
50kDa
30kDa
Flag
50kDa
30kDa
GAPDH
70kDa
50kDa
30kDa
Flag
50kDa
30kDa
GAPDH
ED Figure 6a
70kDa
50kDa
PARP1
70kDa
50kDa
Flag
25kDa
15kDa
Cl.Casp3
25kDa
15kDa
Cl.Casp7
GAPDH
50kDa
30kDa
ED Figure 6b
70kDa
50kDa
Flag
70kDa
50kDa
p-MLKL
70kDa
50kDa
MLKL
70kDa
50kDa
RIPK3
50kDa
30kDa
GAPDH
ED Figure 6c
70kDa
50kDa
30kDa
Flag
70kDa
50kDa
Cl.Gasdermin D
30kDa
30kDa
25kDa
Cl.Casp1
30kDa
25kDa
Cl.IL1-β
50kDa
GAPDH
30kDa
ED Figure 6d
p62/SQSTM1
70kDa
50kDa
70kDa
50kDa
Flag
50kDa
GAPDH
30kDa
15kDa
LC3
ED Figure 8C
50kDa
30kDa
GAPDH
TRF2
70kDa
50kDa
50kDa
30kDa
Flag
ED Figure 8e
TRF2
70kDa
50kDa
30kDa
GAPDH
50kDa
30kDa
Flag
50kDa
30kDa
Flag

SI Figure 4

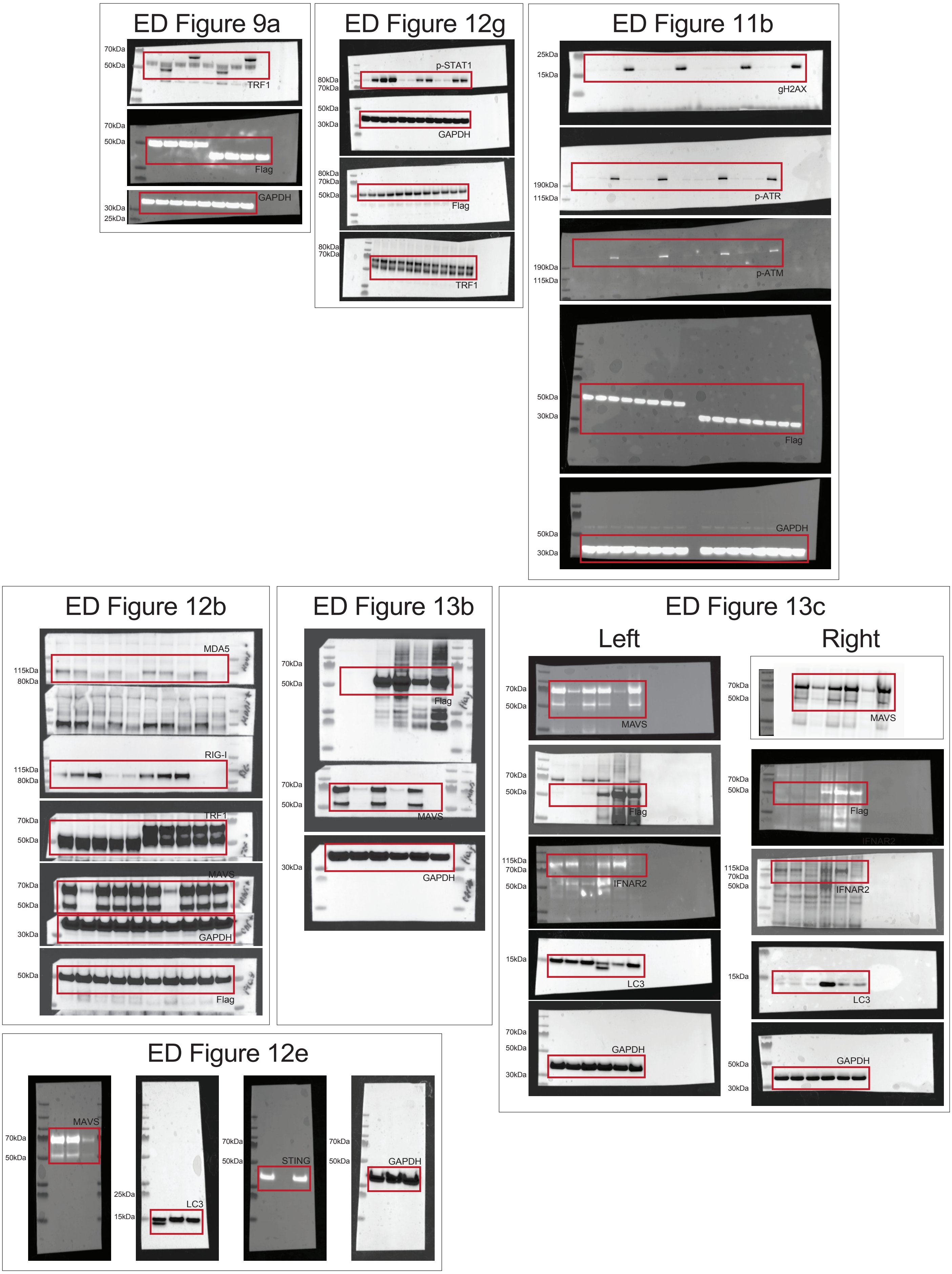
ED Figure 9a
70kDa
50kDa
TRF1
Flag
GAPDH
30kDa
25kDa
ED Figure 12g
p-STAT1
80kDa
70kDa
50kDa
30kDa
GAPDH
Flag
TRF1
ED Figure 11b
25kDa
15kDa
gH2AX
190kDa
115kDa
p-ATR
p-ATM
Flag
GAPDH
ED Figure 12b
MDA5
115kDa
80kDa
RIG-I
TRF1
MAVS
GAPDH
Flag
ED Figure 13b
70kDa
50kDa
Flag
MAVS
30kDa
GAPDH
ED Figure 13c
Left
Right
MAVS
Flag
IFNAR2
LC3
GAPDH
15kDa
ED Figure 12e
MAVS
LC3
STING
GAPDH

SI Figure 5

ED Figure 8a

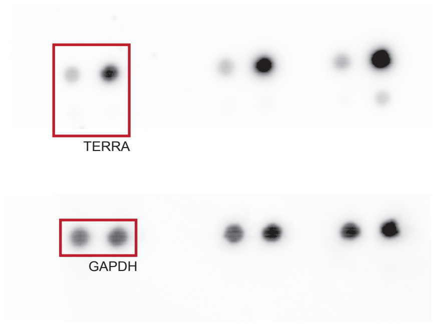

ED Figure 8b

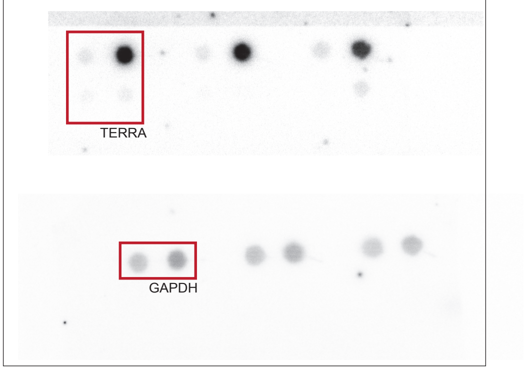

ED Figure 10f

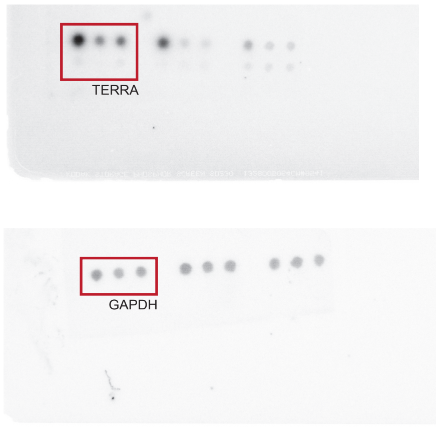

Figure 3d

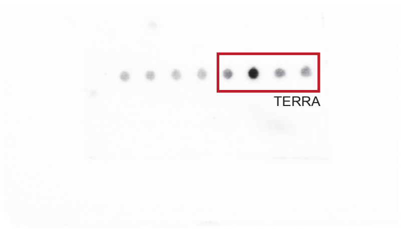

ED Figure 9b

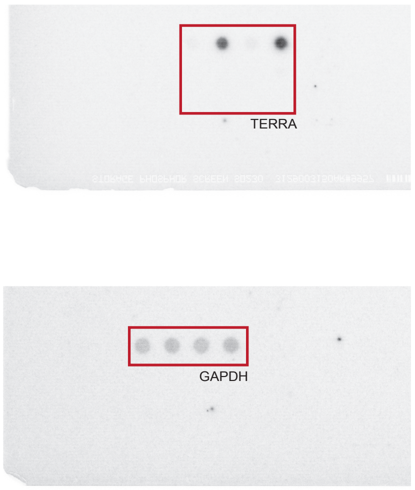

ED Figure 7b

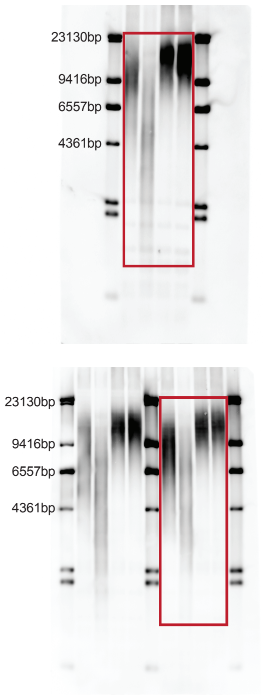

SI Figure 6

List of enriched gRNAs with a log2 fold change > 2 in both replicates (n=170)

sgRNA	FC (Replicate 1)	FC (Replicate 2)	Gene
BRUGEN23565	16.52924271	13.4438975	KALRN
BRUGEN51482	16.28180204	14.01981645	ZBP1
BRUGEN60319	15.97886721	17.17969	STXBP5
BRUGEN39230	13.99489956	9.222125505	CDK5RAP1
BRUGEN64293	13.21393925	9.919663583	FAM9B
BRUGEN69479	11.56455467	18.08529288	PALM3
BRUGEN22154	11.0617839	10.21406082	LIPF
BRUGEN14706	10.65257814	22.90625333	PRB1
BRUGEN09775	10.53436067	5.467736241	ITGAD
BRUGEN25179	10.49719549	4.230500274	CXCL14
BRUGEN07027	10.30110065	10.45419417	GDF2
BRUGEN45823	10.15510525	11.33384257	LRRC7
BRUGEN66268	10.09817923	11.04588281	TECRL
BRUGEN30228	9.810699591	9.98434783	SLC7A9
BRUGEN73171	9.359989352	7.741076113	CDNF
BRUGEN07825	9.129640913	4.626015919	CXCL3
BRUGEN11663	9.022051094	5.542893942	MNT
BRUGEN09705	8.72627877	4.668059439	IRF3
BRUGEN16971	8.47484659	5.750428534	CCL4
BRUGEN57397	8.334062999	4.731074347	MB21D1
BRUGEN27931	8.303823523	5.813952307	NOD1
BRUGEN45664	8.283263224	4.288806119	MAVS
BRUGEN69100	8.240559202	8.412907163	ZC3H12D
BRUGEN33183	8.188079984	6.847104281	CHST5
BRUGEN28062	8.021982733	7.941816086	RBM14
BRUGEN50930	8.020777167	6.058754354	ALPK1
BRUGEN41636	7.891255188	4.338897385	AKIRIN2
BRUGEN29828	7.818190743	4.117045154	IL24
BRUGEN69081	7.77598133	8.245880665	TMEM173
BRUGEN34475	7.773342807	8.820661865	ANKRD17
BRUGEN08051	7.554932377	8.094777681	GYS1
BRUGEN51484	7.488900056	11.749319	ZBP1
BRUGEN09721	7.353741853	4.887147568	IRF7
BRUGEN09453	7.340992486	8.404800552	CXCL8
BRUGEN09252	7.155325433	5.879855491	IFNAR1
BRUGEN28502	6.788540577	4.073442412	CXCL13
BRUGEN16973	6.775748232	5.128161819	CCL5
BRUGEN28588	6.658822756	5.587697823	MTHFS
BRUGEN22954	6.655594275	5.226231396	RIOK3
BRUGEN28845	6.639821784	4.961666643	CXCR6
BRUGEN17011	6.591877302	4.266515897	CCL18
BRUGEN01604	6.590936434	5.424043065	BBS4
BRUGEN09681	6.519130997	4.53211718	IRAK1

BRUGEN67759	6.506612866	5.166281814	CXCL17
BRUGEN02600	6.502673895	4.14042414	CD14
BRUGEN28571	6.501314625	4.522773061	IFITM2
BRUGEN27582	6.435440823	4.38235593	AKAP8
BRUGEN17005	6.338713233	4.580522641	CCL17
BRUGEN11352	6.309526607	4.772985803	MAP3K5
BRUGEN28162	6.185425562	5.503667188	CLEC10A
BRUGEN60177	6.122106099	4.157170456	PRRC1
BRUGEN09258	6.102840283	5.546911167	IFNAR2
BRUGEN26236	5.884771601	6.105015639	TRIM66
BRUGEN16964	5.866982748	4.319532542	CCL3
BRUGEN74970	5.859263836	5.616503281	CCDC175
BRUGEN09612	5.751167402	6.613801853	CXCL10
BRUGEN42573	5.748773268	5.562717757	SLC35E3
BRUGEN39540	5.711998091	4.346836294	IL17D
BRUGEN04489	5.708095929	4.730652913	DGKA
BRUGEN54665	5.691036763	4.87634195	DISP1
BRUGEN55100	5.685274031	5.508521688	SERPINB11
BRUGEN20683	5.65599792	6.669538741	ZNF138
BRUGEN42695	5.623909341	6.899744139	STAB2
BRUGEN17049	5.623116429	5.604071255	CXCL5
BRUGEN07584	5.585939944	6.246052457	GPER1
BRUGEN72587	5.450410556	4.364483663	IFITM10
BRUGEN13276	5.409217236	5.815723608	PAK1
BRUGEN07523	5.39489073	7.856981555	CXCR3
BRUGEN61494	5.375288425	6.872615704	HAPLN3
BRUGEN58271	5.364787675	5.645232685	ARL14EP
BRUGEN72442	5.358151098	6.543153369	C8orf59
BRUGEN22176	5.352584635	4.384983137	IFITM1
BRUGEN60514	5.343059705	6.99341914	ADHFE1
BRUGEN68496	5.333501187	6.849608718	TRIM59
BRUGEN05316	5.317519925	4.68391798	EEF1D
BRUGEN42139	5.311239709	6.15613453	PSPC1
BRUGEN62694	5.277219909	4.214827753	MGAT4D
BRUGEN57398	5.272575113	8.993357947	MB21D1
BRUGEN15535	5.225991327	7.318125413	PTX3
BRUGEN20268	5.217643447	6.145506411	WNT5A
BRUGEN25959	5.209394392	6.000216313	MATR3
BRUGEN27184	5.177128069	6.265590048	TRIM28
BRUGEN09372	5.1579951	6.386460475	IL1A
BRUGEN19279	5.133772647	4.877663034	TLR2
BRUGEN71153	5.115351396	4.431856355	LYSMD1
BRUGEN27597	5.080171644	6.713646892	NET1
BRUGEN34502	5.077587996	4.97754299	LSM14A
BRUGEN53409	5.074086591	5.455407666	TMEM246

BRUGEN12692	5.044695113	4.915981395	NONO
BRUGEN00781	5.009071077	7.923322092	ANG
BRUGEN39201	4.995244147	16.11192047	MRPL48
BRUGEN57192	4.995244147	10.74128032	GPRIN1
BRUGEN04480	4.947967787	4.187441515	CD55
BRUGEN62006	4.908832742	6.38237706	SYT6
BRUGEN11492	4.883724954	7.852752914	CXCL9
BRUGEN29060	4.875407403	6.677660305	MASP2
BRUGEN00773	4.834174038	4.242828996	AMY2A
BRUGEN56324	4.811571083	7.211077004	DPH7
BRUGEN06722	4.794176163	6.872615704	GAB1
BRUGEN19286	4.791172144	5.623794799	TLR4
BRUGEN44431	4.785468472	4.546832291	TCEAL7
BRUGEN09498	4.771950447	4.307739196	IL12B
BRUGEN26729	4.749851909	4.530231595	HDAC6
BRUGEN07820	4.746183757	7.700051432	CXCL2
BRUGEN09263	4.733650589	5.033384308	IFNB1
BRUGEN21001	4.698857749	7.894177258	CXCR4
BRUGEN09461	4.691802244	4.052140074	IL9
BRUGEN49424	4.678100567	4.00320118	NDNF
BRUGEN39781	4.6367359	4.370817979	TLR9
BRUGEN73285	4.619382051	4.663694466	BPY2B
BRUGEN12005	4.584449829	4.802382486	MYD88
BRUGEN16982	4.576262523	5.433993616	CCL8
BRUGEN63307	4.575938644	6.719759009	PATE1
BRUGEN28844	4.567223832	8.89326042	CXCR6
BRUGEN07184	4.544518698	4.614595585	GLA
BRUGEN17077	4.534208412	6.123067386	CXCL12
BRUGEN09434	4.516154719	5.639542706	IL6
BRUGEN33833	4.511025547	6.235755685	TXN2
BRUGEN30681	4.497333227	4.566255557	TREX1
BRUGEN52639	4.480408874	5.48002035	TTC29
BRUGEN66242	4.471904807	4.492942886	IL27
BRUGEN51690	4.461378392	6.541556657	TRIM11
BRUGEN16976	4.455646936	5.626015919	CCL7
BRUGEN23854	4.448037362	4.989319015	USP14
BRUGEN03069	4.439304205	7.95486825	CFL2
BRUGEN15674	4.431056033	7.932485377	MAP4K2
BRUGEN32233	4.415817046	6.585475959	KIAA1024
BRUGEN31949	4.40746648	8.195256587	TTLL12
BRUGEN65922	4.39335196	5.609174492	FOXK1
BRUGEN35996	4.377947747	4.360125358	HPGDS
BRUGEN47965	4.359045793	6.248841418	(MARC1)
BRUGEN09466	4.354413076	6.582479257	CXCR2
BRUGEN16984	4.347204556	8.130495921	CCL11

BRUGEN51522	4.338286744	4.370753862	OR8J3
BRUGEN18344	4.33369833	4.931411434	STAT2
BRUGEN66985	4.326119026	5.2655432	TAS2R41
BRUGEN20318	4.318299222	4.408336381	WRN
BRUGEN42458	4.31395358	4.424081671	HEMGN
BRUGEN32076	4.311579951	4.01751838	PLEKHM2
BRUGEN16696	4.301963885	4.162733469	RYR1
BRUGEN57375	4.297221399	5.537639209	TSGA13
BRUGEN36513	4.263402827	4.630018096	TBK1
BRUGEN08480	4.245193337	4.023765651	HMGB2
BRUGEN57999	4.239444492	4.705488608	TRIM6
BRUGEN26510	4.231429294	4.325681928	EXOG
BRUGEN08344	4.207225867	4.379887722	UBE2K
BRUGEN76959	4.205292608	12.43363886	Non-Targeting_Control
BRUGEN09422	4.199279366	6.477839853	ANKLE1
BRUGEN06715	4.196392916	7.375381295	XRCC6
BRUGEN17040	4.189562907	5.372894241	CXCL6
BRUGEN46545	4.176372227	4.14979764	NLRC4
BRUGEN25455	4.172513429	8.933798974	IKBKE
BRUGEN36389	4.163236727	7.81564441	SETD2
BRUGEN12963	4.147726078	4.949214213	OAS2
BRUGEN62535	4.13990361	4.744648999	DTX3L
BRUGEN31326	4.135130841	8.823085216	TRIM32
BRUGEN73674	4.128325618	4.965182359	FAM110C
BRUGEN70828	4.118736198	4.603383625	IFITM5
BRUGEN49584	4.115239073	5.359715015	NLRX1
BRUGEN23131	4.110755172	5.037214487	CD84
BRUGEN11618	4.096257661	5.054574576	MMP12
BRUGEN59369	4.090493288	5.414217259	HMGB4
BRUGEN27992	4.087194785	4.195464085	IFITM3
BRUGEN54836	4.058355003	8.060707249	ZCCHC3
BRUGEN18341	4.05426138	5.742631276	STAT1
BRUGEN72006	4.042347568	6.172768253	USP17L8
BRUGEN09430	4.023658953	4.601343393	IL5RA
BRUGEN11553	4.021620506	4.483990555	AFF1
BRUGEN01750	4.020242142	6.24117984	CXCR5
BRUGEN59979	4.014569383	7.399314883	TMEM42
